# The comparison of general health status between hemodialysis and kidney transplant patients in university hospitals of Ahvaz, Iran

**DOI:** 10.12861/jrip.2014.09

**Published:** 2013-11-03

**Authors:** Hatam Boostani, Ali Ghorbani, Maryam heydarazadzadeh

**Affiliations:** ^1^Department of psychiatry, Golestan Hospital, Ahvaz Jundishapur University of Medical Sciences, Ahvaz, Iran; ^2^Department of Nephrology, Golestan Hospital, Ahvaz Jundishapur University of Medical Sciences, Ahvaz, Iran

**Keywords:** Hemodialysis, Intima–media thickness, End-stage renal disease

## Abstract

**Introduction:** Patients undergoing chronic hemodialysis and kidney transplant patients will be afflicted with various mental and physical problems, which may affect their general health. Previous studies have shown that, general health level in these patients is lower than general population. However, definitive studies comparing the general health between the two groups of patients undergoing hemodialysis and kidney transplant recipients have not been done, yet.

**Objectives:** In this study, we aimed to compare the general health between the two groups of chronic hemodialysis patients and kidney transplant recipients.

**Methods and Patients:** This investigation was a cross-sectional descriptive-analytic study that has been conducted on 31 hemodialysis and 33 kidney transplant patients in two university hospitals of Ahvaz, Iran. Data were collected through a general health questionnaire (GHQ-28) and they were analyzed with SPSS software.

**Results:** The mean score of general health was 28.8 and 27.57 in hemodialysis and kidney transplant patients, respectively. There was no statistically significant difference between the two groups, (p =0.7). Also, anxiety, serious depression, physical symptoms and social function were not significantly different between the two groups (p>0.5).

**Conclusion:** In this study, no difference of general health level between hemodialysis and kidney transplantation was observed.

**Implication for health policy/practice/research/medical education:**

In a study on patients undergoing chronic hemodialysis and kidney transplant recipients, we compared the general health between the two groups. We found that, there is no clear difference between kidney transplant recipients and chronic hemodialysis patients at the level of general health and amount of depression, anxiety, physical symptoms and social function.

## Introduction

Chronic and severe kidney dysfunction can cause many physical and mental problems. As disease progresses, accumulation of toxins will reach to an extent that patients usually experience a significant influence in their daily activities including feeling of health and nutrition status. This condition may lead to death unless renal replacement therapy (dialysis or kidney transplantation) is started (1).

Due to great efforts to reduce mortality and morbidity in these patients, the number of these patients is increasing considerably (2). However, patients with end-stage renal disease (ESRD) can still have a lot of psychological problems.

The fact that patients survival depends on a chronic dialysis, has a large stress. Indeed, this dependency to the dialysis machine at any time may cause a mental dysfunction. In kidney transplanted patients, there are several problems that may lead to a disorder in the quality of life and their general health such as rejecting the graft, side effects of immunosuppressive medications (particularly neurological/psychological effects) and the probability of disease recurrence (3,4). In some dimensional studies, the general health between the two groups of hemodialysis and kidney transplant patients has been compared and different results have been reported.

## Objectives

According to the above-mentioned problems and the lack of related studies in the country, we conducted a comparative study between general health level, anxiety, depression, physical and social function in these two groups of patients in Imam and Golestan Hospitals in Ahvaz. We specified areas that are less influenced from the above modalities beside the investigation and comparison of kidney alternative therapeutic options on general health. Also, we tried to find a solution for a higher general health for these patients along with decision making for the better kind of alternative modality.

## Patients and Methods

This cross-sectional study was a descriptive-analytic investigation.

### Patients

The participants were selected as conveniently available among patients undergoing chronic hemodialysis (dialysis duration > 6 months) and kidney transplant recipients (grafted > 6 months ago and serum creatinine ≤ 2 mg/dl) that referred to Imam and Golestan hospitals of Ahvaz. They were matched in variables such as age, sex and marital status. Age above 18 and below 65 years and having no history of psychiatric illness were other inclusion criteria. Thirty one hemodialysis patients (16 males) and 33 kidney transplant recipients (18 males) were enrolled in the study. In order to assess the general health, the general health 28-item questionnaire (GHQ-28), the most recognized screen tool in psychiatry, was used and it was designed to screen non-psychotic mental disorders by Goldberg and Hiller in 1979. In Iran, Palahang (5) and Yaghoubi (6) have estimated the test validity and reliability about 91% and 88%, respectively. The questionnaire included 28 articles and 4 subscales (each includes 7 articles) as following:

1. Physical symptoms, 2. Anxiety, 3. Social function, 4. Serious depression.

Every question was scored by Likert scale from 0 to 3 (score extent of each range was from 0 to 21 and a total score obtained by adding up the scores). In several previous studies in Iran, the cut-off point of the questionnaire was determined at 21-23 (7). The cut-off point of 23 was used in this study. In the total test, the score higher than 23 indicated mental disorders and the score lower than 23 was considered as mental disorder. Also, cut-off point of 6 was used in investigating subscales. After taking informed consent from patients, the questionnaires were handed out. If the patients were unable to complete the questionnaire, the researcher completed it for them.

### Ethical issues

(1) The research followed the tenets of the Declaration of Helsinki (2); informed consent was obtained (3); the research was approved by ethical committee of Ahvaz Jundishapur University of Medical Sciences.

### Statistical analysis

Descriptive statistics including mean, standard deviation, minimum and maximum scores and inferential statistics including the comparison of the two groups were done through *t*-test. Data were analyzed by SPSS version 14 and p<0.05 was considered statistically significant.

## Results

The mean scores of general health in the kidney transplant recipients and patients under hemodialysis were 27.57 and 28.8, respectively. According to p=0.7, the differences of general health level between the two groups were not significant (Table 1 ). Also, based on the age division between males and females in transplant recipients (p=0.71) and in hemodialysis patients (P=0.64), no significant difference was observed. The mean score of depression in transplant patients was 3.57 and that of hemodialysis patients was 3.22 (p=0.8). Thus, there was no significant difference between the two groups (Table 1 ). The mean score of anxiety in transplant and hemodialysis patients were 7.45 and 7.41 scores, respectively. In this case, there was no significant difference between the two groups (p=0.9). The mean scores of physical symptoms in transplant and hemodialysis patients were 7.68 and 7.99, respectively, according to p=0.7, there was no significant difference between two groups. The mean scores of social function of transplant and hemodialysis participants were 7.68 and 7.99, respectively and according to p=0.7, no significant difference was detected. The mean scores of social function of transplant and hemodialysis patients were 8.84 and 10.06, respectively (p=0.2), no statistically significant difference was found. The minimum amount of disorder in subscales between the two groups (males and females) was the serious depression that its mean score was lower in both groups from the intended cut-off score (8). But in other subscales, the mean scores of patients were more than the intended cut-off score that indicates the unfavorable situation of patients in those areas. In side findings, there was no significant relationship between the duration of transplantation and general health (p=0.46) or the duration of dialysis (p=0.58), and general health in patients. This fact was true for the subscales of general health in both groups (Table 2).

**Table 1:** The mean score of depression in transplant patients and hemodialysis patients

Compared variables	T	df	p	Mean difference
General health in transplant and hemodialysis patients	-0.31	62	0.7	-1.23
Depression in transplant and hemodialysis patients	0.2	62	0.8	0.35
Anxiety in transplant and hemodialysis patients	0.05	62	0.9	0.04
Amount of physical symptoms of transplant and hemodialysis patients	0.28	62	0.7	-0.31
Social function of transplant and hemodialysis patients	-1.14	62	0.2	-1.22

**Table 2:** Correlation coefficient between the duration of receiving transplant, hemodialysis and general health and its subscales.

**Dependent Variable**	**r**	**P**
Duration of transplantation and general health	0.011	0.9
Duration of transplantation and Physical function	0.03	0.8
Duration of transplantation and anxiety	0.015	0.9
Duration of transplantation and social function	0.03	0.87
Duration of transplantation and depression	-0.27	0.88
Duration of hemodialysis and general health	0.19	0.28
Duration of hemodialysis and Physical function	-0.17	0.34
Duration of hemodialysis and anxiety	-0.19	0.29
Duration of hemodialysis and social function	0.21	0.26
Duration of hemodialysis and depression	0.29	0.26

## Discussion

The current study showed that there was no significant difference in terms of general health between the transplant and hemodialysis patients. The study of Mohajer *et al*. on patients before and after kidney transplant with the GHQ-28 questionnaire reported consistent results (9). Also, in a study by over Beck *et al*. (10), there was no significant difference between the general health levels of two groups with the GHQ-28 questionnaire. According to the cut-off point of 23 that was considered for the general health of patients in this study, general health is unfavorable both in chronic hemodialysis and kidney transplant patients. This result may suggest that although kidney transplantation has provided more chance of rehabilitation and longevity, it has not a significant impact on patients, general health. Even in some studies, contrary to expectation, kidney transplant recipients have reported a more unfavorable quality of life than chronic hemodialysis patients (5,11).

According to our results, there was no difference between two groups in the area of serious depression. In the study of Karaminia *et al*., the amount of anxiety and depression between 32 transplant patients and 39 hemodialysis patients was compared. Results indicated that anxiety was clearly lower in kidney transplant recipients than hemodialysis patients but no difference was observed between the two groups in the rate of depression (4). Ashkani *et al*. investigated the mental damage before and after grafting in 45 kidney transplant patients. Results showed that participants had a significant improvement in terms of depression, social functioning, physical and mental general health after transplantation but there was no significant improvement in patients’ anxiety status (12). Some studies showed more desirable general health level in transplant recipients (6,7), while in other studies, no difference in general health level of the two groups was found (8,9). In some investigations, contrary to expectation, kidney transplant recipients were reported to have a more unfavorable quality of life than patients undergoing chronic hemodialysis (11). The study of Karaminia *et al*. showed consistent results in serious depression (4). There was no significant difference between two groups in the area of anxiety. Ashkani *et al*. (12) investigated the mental damage in 45 patients who received kidney transplant, before and after grafting. No significant difference has been observed in the improvement of patients’ anxiety level with GHQ-28 questionnaire. No significant difference was observed in transplant and hemodialysis patients in physical symptoms. Also, no significant difference was observed in transplant and hemodialysis cases in the area of social function. This result is in concordance with the results of Reimer *et al*. study (13).

In side findings, we observed no statistically significant difference between the duration of transplantation and hemodialysis with patients’ general health. Also, Navidian *et al*. has shown the same results (14). Although most of the studies confirm the impact of dialysis duration on intensity, extent, and amount of mental problems especially depression (15,16), results of some studies, similar to the results of ours, have shown that although physical function of incurable and chronic patients reduces with time, their mental hygiene status will be constant; this consistency may originates from adaptation process in these patients (14-16).

## Conclusion

The findings of this study showed that, contrary to expectation, no clear difference was observed between kidney transplant recipients and chronic hemodialysis patients at the level of general health and amount of depression, anxiety, physical symptoms and social function. Due to the growing number of patients seeking a renal replacement modality, we suggest that more studies should be focused on different aspects of general health in kidney transplant patients by using special questionnaire in terms of depression, anxiety, and etc. Areas that are less influenced from this modality should be clarified to represent some approaches and to improve the general health of these patients while selecting the type of alternative treatment.

## Acknowledgments

This study was taken from the general medical thesis. The authors would like to thank the research deputy of Ahvaz Jundishapur University of Medical Sciences.

## Authors’ contributions

AG and HB conducted the research.MH and AG wrote the manuscript.

## Conflict of interests

The authors declared no competing interests.

## Ethical considerations

Ethical issues (including plagiarism, informed consent, misconduct, double publication and redundancy) have been completely observed by the authors.

## Funding/Support

This study was funded by Ahvaz Jundishapur University of Medical Sciences. (Grant# A-108)
